# Myeloid-derived suppressor cells and vaccination against pathogens

**DOI:** 10.3389/fcimb.2022.1003781

**Published:** 2022-09-29

**Authors:** Estefanía Prochetto, Eliana Borgna, Carlos Jiménez-Cortegana, Víctor Sánchez-Margalet, Gabriel Cabrera

**Affiliations:** ^1^ Laboratorio de Tecnología Inmunológica, Facultad de Bioquímica y Ciencias Biológicas, Universidad Nacional del Litoral, Santa Fe capital, Argentina; ^2^ Clinical Laboratory, Department of Medical Biochemistry, Molecular Biology and Immunology, School of Medicine, Virgen Macarena University Hospital, University of Seville, Seville, Spain

**Keywords:** MDSCs, myeloid-derived suppressor cells, vaccine, pathogens, immunization, parasites, viruses, bacteria

## Abstract

It is widely accepted that the immune system includes molecular and cellular components that play a role in regulating and suppressing the effector immune response in almost any process in which the immune system is involved. Myeloid-derived suppressor cells (MDSCs) are described as a heterogeneous population of myeloid origin, immature state, with a strong capacity to suppress T cells and other immune populations. Although the initial characterization of these cells was strongly associated with pathological conditions such as cancer and then with chronic and acute infections, extensive evidence supports that MDSCs are also involved in physiological/non-pathological settings, including pregnancy, neonatal period, aging, and vaccination. Vaccination is one of the greatest public health achievements and has reduced mortality and morbidity caused by many pathogens. The primary goal of prophylactic vaccination is to induce protection against a potential pathogen by mimicking, at least in a part, the events that take place during its natural interaction with the host. This strategy allows the immune system to prepare humoral and cellular effector components to cope with the real infection. This approach has been successful in developing vaccines against many pathogens. However, when the infectious agents can evade and subvert the host immune system, inducing cells with regulatory/suppressive capacity, the development of vaccines may not be straightforward. Notably, there is a long list of complex pathogens that can expand MDSCs, for which a vaccine is still not available. Moreover, vaccination against numerous bacteria, viruses, parasites, and fungi has also been shown to cause MDSC expansion. Increases are not due to a particular adjuvant or immunization route; indeed, numerous adjuvants and immunization routes have been reported to cause an accumulation of this immunosuppressive population. Most of the reports describe that, according to their suppressive nature, MDSCs may limit vaccine efficacy. Taking into account the accumulated evidence supporting the involvement of MDSCs in vaccination, this review aims to compile the studies that highlight the role of MDSCs during the assessment of vaccines against pathogens.

## Introduction

The immune system has always been characterized by its effector function related to coping pathogens and anomalous cells. However, accumulating evidence since the 1970s supported the existence of both lymphoid and myeloid cells with regulatory/suppressive capacity (Gershon and Kondo, 1970; Bennett et al., 1978). Since the initial proposal and after decades of research, to date, it is widely accepted the existence of T-cells (Sakaguchi et al., 1995; Shevach, 2006; Plitas and Rudensky, 2016) and non-T cells such as myeloid-derived suppressor cells (MDSCs) with regulatory/suppressive capacity that play a role in almost any process in which the immune system is involved, including infections, cancer, autoimmune disease, allergies, obesity, transplants, pregnancy, vaccination and other scenarios (2021; Nagaraj and Gabrilovich, 2007; Medina and Hartl, 2018; Ostrand-Rosenberg, 2018; Ahmadi et al., 2019; Pawelec et al., 2019; Kidzeru et al., 2021; Veglia et al., 2021).

The terminology of MDSCs was proposed in 2007 (Gabrilovich et al., 2007) to include a heterogeneous population of myeloid origin, an immature state, and a strong capacity to suppress not only T-cells but also other immune populations (Nagaraj and Gabrilovich, 2007; Veglia et al., 2021). Most of the knowledge acquired about these cells was gained by their study in pathological conditions such as cancer and acute and chronic infections, but currently, a large body of evidence supports the involvement of MDSCs also in physiological/nonpathological conditions, such as pregnancy, neonatal period, aging, and vaccination (2021; Ahmadi et al., 2019; Cabrera and Marcipar, 2019; Pawelec et al., 2019; Veglia et al., 2021).

Vaccination is one of the public health measures with the greatest benefits to humanity, preventing major epidemics, morbidity, and mortality (Koff et al., 2013; Domínguez-Andrés et al., 2020; Plotkin, 2020). The worldwide pandemic caused by severe acute respiratory syndrome coronavirus-2 (SARS-CoV-2) is a clear example of the pressure that may be exerted on the health systems when a needed vaccine is not available. Moreover, the lack of a vaccine against SARS-CoV2 not only increased deaths and disease but also forced the governments to employ extreme measures such as quarantines, social distancing, travel restrictions, and other interventions that caused a huge economic and social impact (Milne et al., 2021; Feikin et al., 2022; Goldblatt et al., 2022). Notably, accelerated vaccine research using all old and new strategies of vaccine development allowed to markedly diminish the impacts of the pandemic (Goldblatt et al., 2022).

Vaccines are biological preparations that are used to stimulate the effector arm of the immune system, including humoral and cellular components, to confer protection against infection and/or disease on subsequent exposure to a given pathogen (Koff et al., 2013; Domínguez-Andrés et al., 2020; Plotkin, 2020; Pollard and Bijker, 2021). The strategy based on the stimulation of the effector response has been sufficient in many cases to develop very successful vaccines against many pathogenic microorganisms (Plotkin, 2020). However, there is a long list of pathogens for which there is no available vaccine or that the existent vaccine should be improved (Plotkin, 2018). Interestingly, many if not all of the pathogens for which a vaccine is still lacking are endowed with the capacity to evade and subvert the host immune system, inducing cells with regulatory/suppressive capacity, such as HIV, HCV, *Trypanosoma cruzi* (*T. cruzi*), *plasmodium falciparum*, *Toxoplasma gondii*, *Leishmania* spp., *Mycobacterium tuberculosis*, etc (Van Ginderachter et al., 2010; Goh et al., 2013; Ost et al., 2016; Singh et al., 2016; O’Connor et al., 2017; Dorhoi et al., 2019; Koushki et al., 2021). In addition, since immunization is designed to mimic, at least in a part, certain aspects of the natural infection, it is not illogical that the immunization process generates by itself MDSC increases. For instance, the use of the attenuated Bacillus Calmette-Guerin (BCG) vaccine has been shown to elicit MDSC increases (Martino et al., 2010), a feature that has also been described for *M. tuberculosis* (du Plessis et al., 2013; Magcwebeba et al., 2019; Kotzé et al., 2020), the pathogen for which the BCG vaccine has been developed.

The induction of MDSCs does not depend on a particular pathogen, adjuvant, or immunization route. In contrast, the involvement of the immunosuppressive population has been documented in vaccine studies including viruses, bacteria, parasites, and fungi. Different adjuvants and also immunization routes have been implicated in MDSC increases, clearly supporting the notion that the study of the role played by MDSCs could be valuable in many settings and may affect vaccine efficacy. Moreover, conditions in which vaccine effectiveness may be decreased, such as aging, obesity and newborns are associated with MDSC increases (Chen et al., 2015; Painter et al., 2015; Köstlin-Gille and Gille, 2020; Pawelec et al., 2021).

According to this, the present review aims to compile reports supporting the study of MDSCs as a valuable and complementary tool to develop or improve vaccines against several complex pathogens.

## History of MDSCs in vaccination against pathogens

**Figure 1:**
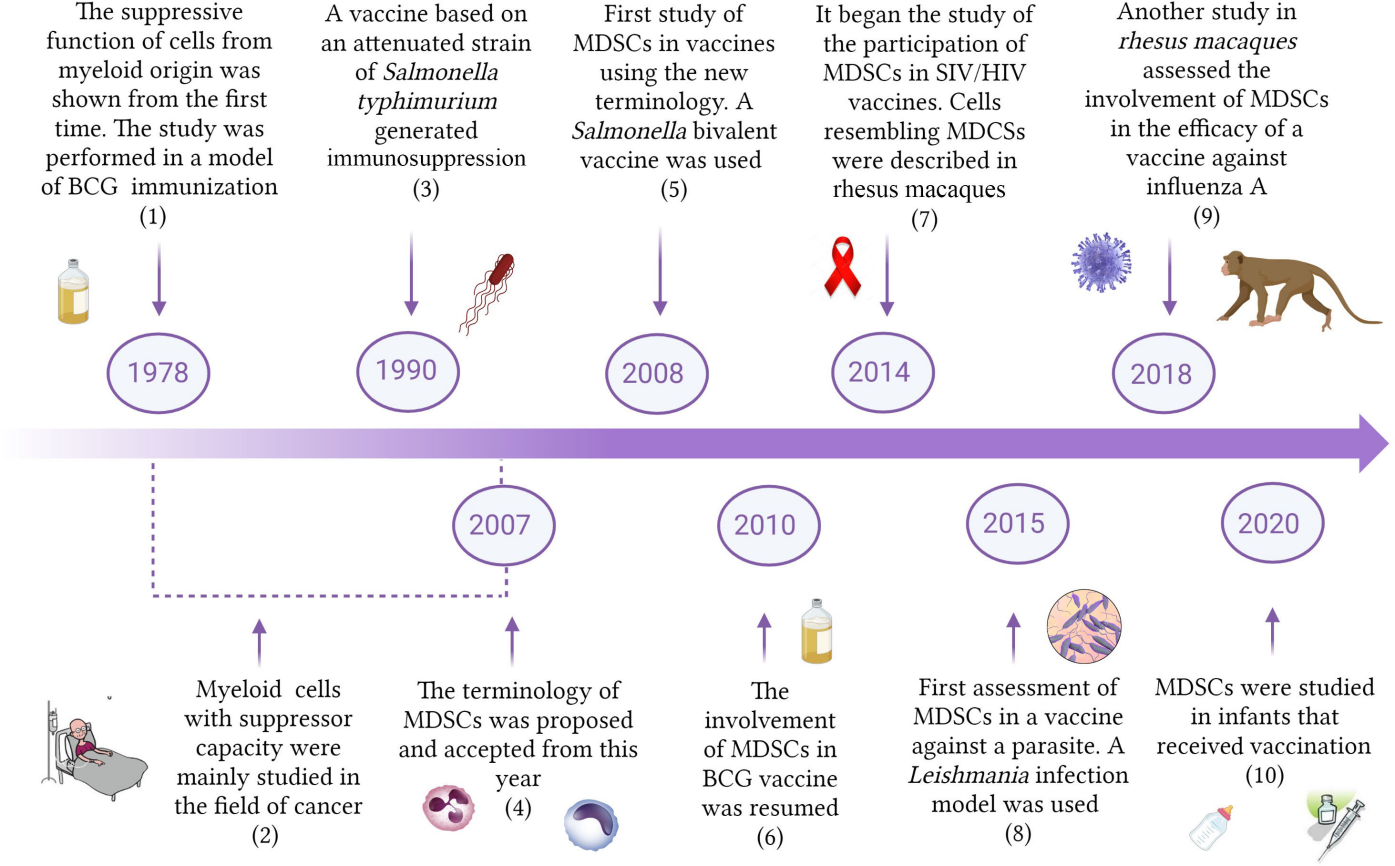
Timeline of the study of MDSCs in vaccination. The Figure shows some important reports describing the involvement of MDSCs in vaccination against different pathogens. BCG, Bacillus Chalmette-Guerin; MDSCs, Myeloid-derived suppressor cells; HIV, Human immunodeficiency virus; SIV, simian immunodeficiency virus. (1) Bennett et al., 1978; (2) Koyama et al., 1982; Yang et al., 2006; (3) al-Ramadi et al., 1991; (4) Gabrilovich, et al., 2007; (5) Heithoff et al., 2008; (6) Martino et al., 2010; (7) Sui et al., 2014; (8) Bandyopadhyay et al., 2015; (9) Lin et al., 2018; (10) Kidzeru et al., 2021.

The first studies providing support to the concept that the immune system included cells whose function would be to regulate and suppress other immune cells were published in the 1970s (Gershon and Kondo, 1970; Bennett et al., 1978; Sakaguchi et al., 2007). Interestingly, one of the pioneer studies demonstrating the suppressive capacity of myeloid cells was carried out in a model of BCG inoculation (Bennett et al., 1978) (**Figure 1**). Bennet et al., 1978 showed that bone marrow natural suppressor cells were activated after systemic administration of BCG. In addition, the treatment also caused suppressor cell increases in the spleen through the migration and colonization of that organ by bone marrow elements (Bennett et al., 1978). Strikingly, the authors stated in 1979 that modulation of immunity by BCG is the vectorial sum of its many effects, augmenting immunity but also suppressive activities that often co-exist with adjuvant effects (Mitchell and Murahata, 1979). This pioneering work was not followed by other studies and the research of the involvement of myeloid cells with suppressive capacity in vaccine studies against pathogens did not receive special attention. In the 1990s it was reported that mice immunized with an attenuated strain of *Salmonella typhimurium* generated protection against a virulent challenge, but exhibited suppressed responses to B and T cell mitogens in the culture of splenocytes, showing a nitric oxide (NO)-dependent immunosuppressed state (al-Ramadi et al., 1991; 1992). This report was also an isolated case, and the study and initial characterization of myeloid cells with suppressive capacity were mainly performed in the field of cancer (Talmadge and Gabrilovich, 2013). Since suppressive myeloid cells do not express classic membrane markers of T cells, B cells, natural killer cells (NKs), or macrophages, studies focalized on this population used different terminologies such as natural suppressor cells, immature myeloid cells or null cells (Talmadge and Gabrilovich, 2013; Li et al., 2021). In 2007, a letter written by D. Gabrilovich et al. proposed the name of myeloid-derived suppressor cells (MDSCs), and a consensus was reached regarding the nomenclature of this population of suppressive cells of myeloid origin (Gabrilovich et al., 2007).

In 2008 it was published the first vaccine study using the new terminology and demonstrating the involvement of MDSCs during immunization with a *Salmonella* bivalent vaccine (Heithoff et al., 2008). Since then, MDSC participation was studied in vaccines designed to immunize against other pathogens including viruses, bacteria and parasites. New interest in the role of MDSCs during the assessment of vaccines against *M. tuberculosis* gave raise to additional reports from 2010 (Martino et al., 2010). The first study concerning the role of MDSCs in SIV/HIV vaccines was published only 8 years ago, in 2014 (Sui et al., 2014). The involvement of MDSCs in vaccine studies against parasites started in 2015, after a work conducted on a model of immunization against *Leishmania* (Bandyopadhyay et al., 2015). Lin et al. reported in 2018 that MDSCs in circulation early increased after influenza A vaccination of rhesus macaques (Lin et al., 2018), which constitutes a very valuable model for late-stage vaccine testing before clinical trials. The relevance of the frequency of MDSCs in infants receiving vaccination began to be studied only 2 years ago. A higher frequency of MDSCs at the time of vaccination was associated with a lower immune response against certain antigens (Kidzeru et al., 2021). The increasing evidence supporting the involvement of MDSCs as an important part of vaccine assays guarantees that further research will be performed next years in this direction.

## Markers and origin of myeloid-derived suppressor cells

**Figure 2:**
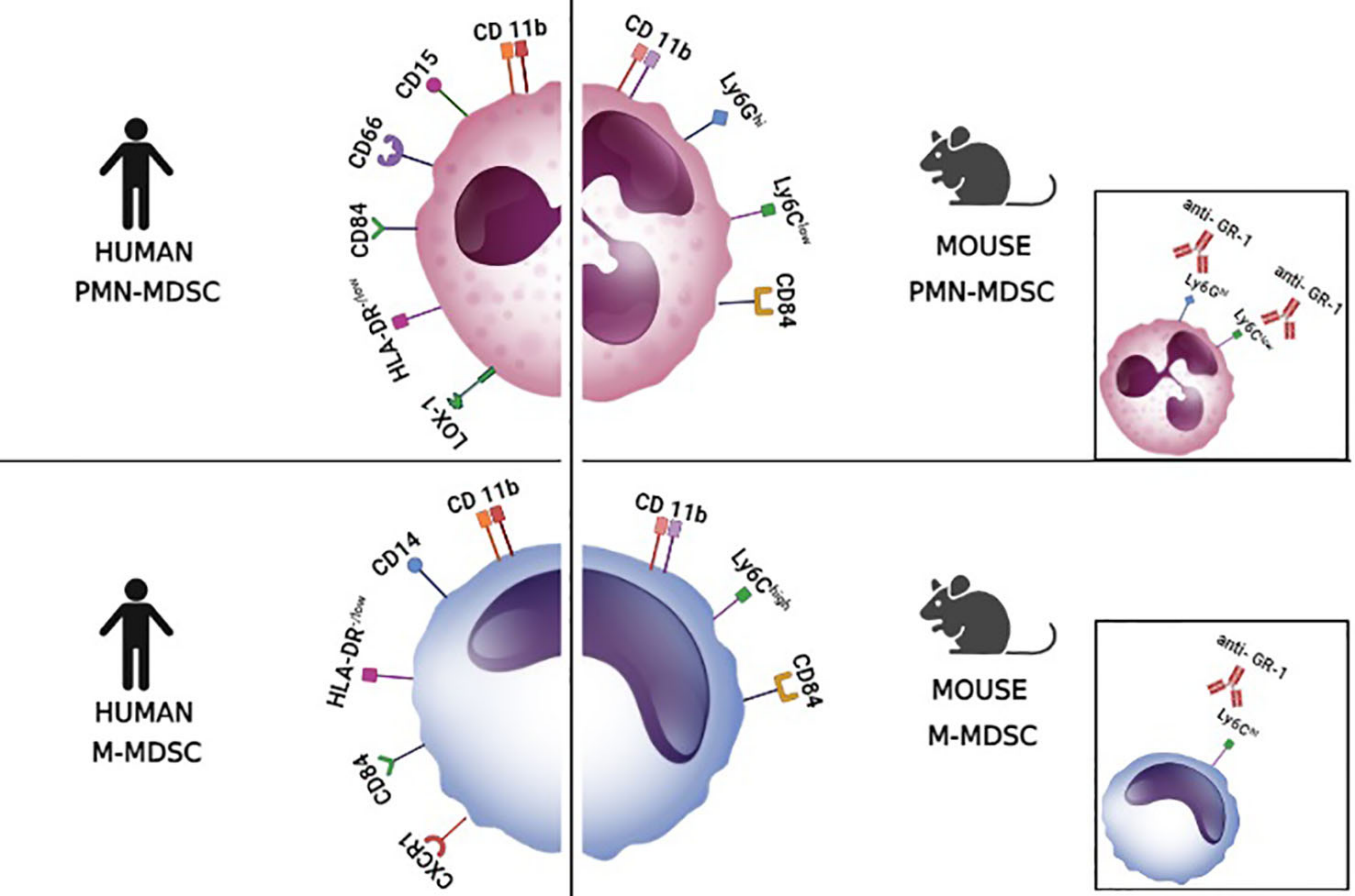
Markers of myeloid-derived suppressor cells. The figure illustrates surface markers of the different subsets of myeloid-derived suppressor cells (MDSCs) in mice and humans. These markers allow the discrimination between polymorphonuclear myeloid-derived suppressor cells (PMN-MDSCs) and monocytic myeloid-derived suppressor cells (M-MDCS). LOX-1, lectin-type oxidized LDL receptor 1+. The small boxes show that the anti-GR-1 antibody binds to both Ly6G and Ly6C molecules.

In mice, there are two main subtypes of MDSCs: M-MDSCs (monocytic MDSCs) which express CD11b+, Ly6G- Ly6C+, and PMN-MDSCs (polymorphonuclear MDSCs, also granulocytic G-MDSCs) which express CD11b+ Ly6G+ and Ly6C^+/low^ (**Figure 2**). In addition, since there is an anti-Gr-1 antibody that binds both Ly6C and Ly6G, in some studies MDSCs are marked as CD11b+ GR-1+ which marks MDSCs but does not differentiate between M-MDSCs and PMN-MDSCs (Bergenfelz and Leandersson, 2020; Veglia et al., 2021). Recently, CD84 has been suggested as an additional marker for MDSCs that are present in the tumor microenvironment (Veglia et al., 2021). In humans, there are no molecules equivalent for Ly6C and Ly6G and thus anti-GR-1 staining is not possible. Instead, the equivalent to M-MDSCs is defined by CD11b+ CD33+ CD14+ CD15- HLA-DR ^-/low^ staining and PMN-MDSCs are defined by CD11b+ CD33+ CD14- CD15+/CD66b+ HLA-DR ^-/low^ (**Figure 2**) (Ostrand-Rosenberg and Fenselau, 2018; Jiménez-Cortegana et al., 2021; Veglia et al., 2021). Similar to mice, the use of CD84 has also been suggested for distinguishing human MDSCs from classical myeloid cells without suppressor capacity. In addition, for differentiating PMN-MDSCs from neutrophils, the staining of lectin-type oxidized LDL receptor 1+ (LOX1+) was proposed. Alternatively, since PMN-MDSCs have low density, the suppressor cells could be separated from neutrophils in a density gradient (1.077 g/ml) (Veglia et al., 2021). Regarding M-MDSCs, it has been suggested that CXCR1+ staining would be useful to distinguish M-MDSCs from classical monocytes (Bronte et al., 2016).

Irrespective of the staining of the markers, it is always recommended to test the function of the potential MDSCs, since the suppressive capacity is considered the hallmark of this population. The inhibition of T-cell proliferation is the most widely used assay to confirm the nature of cells resembling MDSCs (Gabrilovich and Nagaraj, 2009; Bronte et al., 2016; Solito et al., 2019; Veglia et al., 2021).

Under steady-state, growth factors including GM-CSF, G-CSF and M-CSF induce the differentiation of granulocytes and monocytes from myeloid cells in the bone marrow (Barreda et al., 2004; Gabrilovich and Nagaraj, 2009; Millrud et al., 2016). However, under specific settings, the kinetic of production of growth factors, together with increases of other molecules may lead to MDSC expansion. For instance, in a setting of chronic inflammation, or during severe and acute infection, many myeloid populations may be consumed and then the bone marrow may activate an *emergency myelopoiesis* to replenish the consumed cells (Panopoulos and Watowich, 2008; Takizawa et al., 2012; Millrud et al., 2016; Loftus et al., 2018; Veglia et al., 2018). In addition, it has been described that extramedullary myelopoiesis may also play a role in MDSC expansion and activation in cancer and other settings (Song et al., 2005; Millrud et al., 2016; Dietrich et al., 2021).

It has been proposed that the activation and accumulation of MDSCs occur in two partially overlapping phases (2 signals model). Signal 1 leads to the expansion of myeloid cell, which takes place in the bone marrow and spleen; and signal 2 leads to the conversion of immature cells including granulocytic and monocytic cells into activated MDSCs, which takes place primarily in peripheral tissues. The first group of signals necessary for the expansion of immature myeloid cells includes not only growth factors GM-CSF, G-CSF and M-CSF, but also other molecules such as IL-6, IL-1ß, PGE2, TNFα, VEGF, S-SCF, Gal-1, Gal-3 and polyunsaturated fatty acids (Blidner et al., 2015; Veglia et al., 2018; Dorhoi et al., 2019; Fultang et al., 2020). The second group of signals is related to the activation of the cells and includes danger-associated molecular patterns (DAMPs) and inflammatory cytokines such as IFN-γ, IL-1β, IL-4, IL-6, IL-13, PGE2, HMGB1, TNFα and TLR ligands (Parker et al., 2014; Condamine et al., 2015; Fultang et al., 2020; Veglia et al., 2021). Several transcription factors and signaling pathways have been described to participate in the expansion and activation of MDSCs and have been reviewed elsewhere (2021; Condamine et al., 2015; Gabrilovich and Nefedova, 2015; Millrud et al., 2016; Veglia et al., 2018; Fultang et al., 2020).

## MDSC mechanisms of suppression

**Figure 3:**
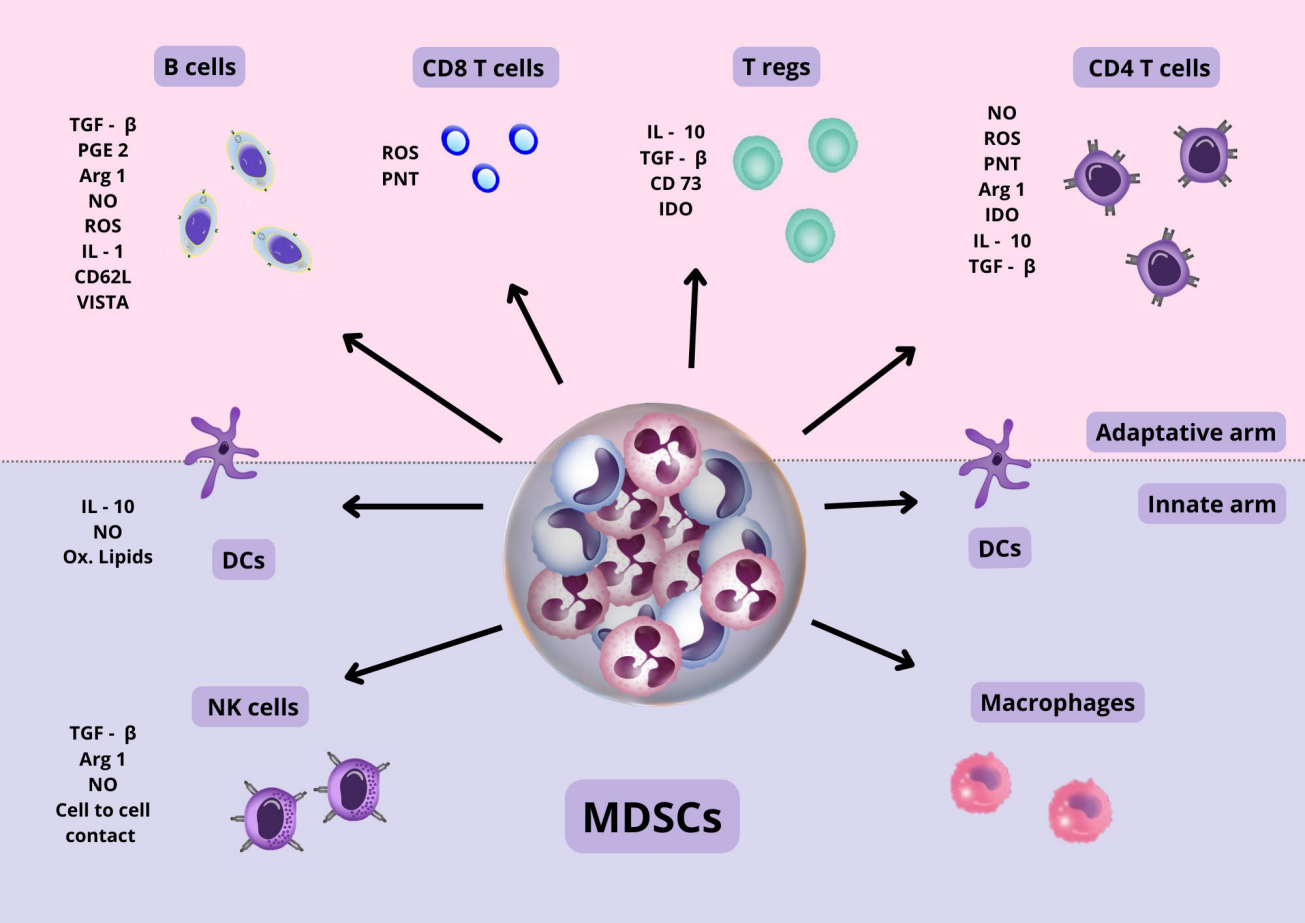
MDSC suppressive mechanisms. MDSC cells, through cell-to-cell contact and the secretion of various anti-inflammatory molecules, produce the suppression of various types of immune cells, both of the innate and adaptive arms. Also, they can affect the function of various proteins and cell migration. The suppression mechanisms of MDSCs are diverse and can exert their action in a specific as well as non-specific manner, as explained in the text. Abbreviations: TGF β, Transforming growth factor β; PGE 2, Prostaglandin E2; NO, Nitric oxide; ROS, Reactive oxygen species; IL-1, Interleukin 1; IL-10, Interleukin 10; CD62L, Cluster of differentiation 62 ligand; CD 73, Cluster of differentiation 73; IDO, Indolamine 2,3-dioxygenase; ARG 1, Argininie1; PNT, peroxynitrite; VISTA, domain Ig suppressor of T cell activation.

Cells of both the innate and adaptive immune response can be suppressed or affected by MDSCs (**Figure 3**). The mechanisms used by MDSCs include A) Production of inhibitory cytokines with suppressor capacity such as IL-10 and free and membrane-bound TGF-β (Li et al., 2009; Yang et al., 2020), B) production of NO by iNOS and reactive oxygen species (ROS) by NADPH oxidase-2) (Yang et al., 2020; Veglia et al., 2021). NO is a second messenger that together with ROS may form peroxynitrites (PNT), a reactive molecule that causes nitration of tyrosine affecting the function of several proteins. In addition, peroxynitrites can also affect the migration of T cells since PNT can cause nitration and inactivation of CCL2 chemokine, which is important for the migration of T cells (Yang et al., 2020; Veglia et al., 2021), C) depletion of amino acids, including: tryptophane consumption by indoleamine-2,3-dioxygenase (IDO), depletion of cystine, which causes a decrease in the precursor needed to produce cysteine (Srivastava et al., 2010), and depletion of arginine by the enzymes arginase-1 and iNOS (Veglia et al., 2018; Davies et al., 2019); D) expression of several molecules in the surface of MDSCs, such as: PD-L1 (Lu et al., 2016; Nam et al., 2019; Yang et al., 2020), VISTA (Deng et al., 2019), CTLA-4, CD80 (Yang et al., 2006), CD40 (Pan et al., 2010); E) production of CCR5 ligands that may recruit Foxp3+ regulatory T cells (Tregs) (Schlecker et al., 2012); F) Synthesis of prostaglandin E2 (PGE2) (Veglia et al., 2018), G) induction of heme oxygenase-1 (HO-1) (De Wilde et al., 2009); H) production of exosomes (Ostrand-Rosenberg and Fenselau, 2018); I) transfer of oxidatively truncated lipids (Ugolini et al., 2020), J) shedding of the CD62L from lymphocytes *via* ADAMS17 (Hanson et al., 2009), K) generation of adenosine *via* CD39 (nucleoside triphosphate diphosphohydrolase) and CD73 (exto-5´nucleotidase) (Ryzhov et al., 2011),

MDSCs may suppress in a non-specific but also specific manner. It has been described that MDSCs can uptake and present the antigenic epitopes to antigen-specific CD8+ cells (Kusmartsev et al., 2005). ROS and peroxynitrites from close MDSCs can nitrate tyrosines in the T-cell receptor and can abrogate the expression of the T-cell receptor (TCR) z-chain (Yang et al., 2020), in both cases impairing that CD8 T cells could interact with Major histocompatibility complex I (MHCI) expressing cells (Nagaraj and Gabrilovich, 2007; Qu et al., 2012). In addition, MDSCs in close contact with target cells may result in down-regulation of L-selectin on naive T cells, interfering with their ability to migrate to their activation site (Hanson et al., 2009). On the other hand, it has been described that specific suppression of CD4+ cells is also possible when sufficient MHC II expression is achieved by MDSCs (Nagaraj et al., 2012). Depletion of cysteine is one of the mechanisms that have been postulated to be involved in this type of suppression (Yang et al., 2020).

Interestingly, It has been described that MDSCs may cause Tregs expansion, mainly in the context of cancer, but there are reports describing that MDSCs are also able to suppress CD4+ Foxp3+ differentiation from CD4 naïve T cells *via* ROS and the IDO enzyme (Centuori et al., 2012; Ji et al., 2016).

It has been postulated that each subset of MDSCs may suppress using some of the mechanisms described, according to the setting (Gabrilovich, 2017; Veglia et al., 2018). PMN-MDSCs would mainly employ ROS and PNT (Raber et al., 2014; Yang et al., 2020; Veglia et al., 2021), whereas M-MDSCs would mainly use arginase-1, NO and suppressive cytokines (Raber et al., 2014; Yang et al., 2020; Veglia et al., 2021).

## MDSCs in vaccination against bacteria

Taking into account the signals that are required to expand and activate MDSCs and considering that vaccination aims to mimic at least some of the events that take place during a given acute infection, it is reasonable to expect that prophylactic vaccination may affect the MDSC population. Currently, there is substantial support showing that the regulatory immune system plays a major role during *Mycobacterium tuberculosis* infection (Cardona and Cardona, 2019; Magcwebeba et al., 2019; Kelly and McLoughlin, 2020). Moreover, the involvement of MDSCs has been described in acute and chronic infection (du Plessis et al., 2013; Knaul et al., 2014; Daker et al., 2015; Magcwebeba et al., 2019; Kotzé et al., 2020). Although BCG is a related and attenuated vaccine, the influence of this bacteria on myeloid cells with suppressive capacity was the first to be shown more than 40 years ago (Bennett et al., 1978) (**Supplementary Table I**). After the consensus on the MDSC terminology, a work by Martino et al. (2010) showed that BCG immunization in a murine model recruited MDSCs to the site of inoculation. The CD11b+Ly-6C^int^Ly-6G^-^ cells produced NO, but did not kill phagocyted BCG and impaired T cell priming in the draining lymph node (Martino et al., 2010). Interestingly, the authors highlighted that a deeper knowledge of the innate immune response elicited by BCG may be necessary to understand why BCG does not consistently protect adults against pulmonary disease. For this purpose, several new vaccine candidates are under study based on new tuberculosis (TB) antigens. Interestingly, it has been suggested that different antigens may not solve the problem and next-generation anti-TB vaccine types should also consider the regulatory arm of the immune system, controlling MDSC influx and maturation to increase immune-priming efficacy (Schaible et al., 2017; Kelly and McLoughlin, 2020).

The association between MDSC-resembling cells and TB received further support in 2007, when it was reported that splenocytes from complete Freund adjuvant (CFA) primed-mice contained suppressive Gr-1+ cells, including monocytic NO-producing Ly6G- cells and granulocytic O2- producing Ly6G+ cells (Dietlin et al., 2007). In a recent report, Ribechini et al. (2019) showed that a vaccine based on heat-killed TB induced spleen M-MDSCs that can be activated to kill dendritic cells (DCs), an additional mechanism that may help to explain the difficulties found to develop a very successful anti-TB vaccine (Ribechini et al., 2019). In this sense, the authors suggested that the involvement of MDSCs in vaccines against *M. tuberculosis* should be investigated in clinical trials (Ribechini et al., 2019). To date, several candidates have been suggested to modulate MDSCs and improve anti-TB vaccine efficacy (Jayashankar and Hafner, 2016).

Despite the role of the regulatory arm of the immune system in vaccine studies received almost no attention during the previous century, in the 1990s it was reported that immunization with an attenuated strain of *Salmonella Typhimurium* induced a marked state of immunosuppression in B and T cell splenocytes cultured with mitogens. Interestingly, suppression was shown to be NO-dependent, a mechanism that currently is very linked to MDSCs (1992; al-Ramadi et al., 1991). Moreover, in 2008, once the MDSC terminology was established (Gabrilovich et al., 2007), Heithoff et al. (2008) showed that a *Salmonella* Bivalent vaccine (Dam) was able to generate cross-protective immunity against several *Salmonella* strains, at the same time that diminished the expansion of MDSCs, as compared with an AroA *Salmonella* strain that induced significant increases of MDSCs (Heithoff et al., 2008). Interestingly, lower levels of MDSCs correlated with increased levels of cross-reactive opsonizing antibodies and increased number of IFN-ɣ-producing memory CD4 and CD8 T splenocytes, alterations which may account for the protection observed in *Salmonella* bivalent vaccinated mice (Heithoff et al., 2008). Based on these results, the authors suggested that interventions capable of reducing MDSCs may be beneficial to enhancing B and T cell stimulation by vaccines (Heithoff et al., 2008).

## MDSCs in vaccination against viruses

Despite HIV was described in the 1980s (Montagnier, 2010), a prophylactic or therapeutic vaccine is not yet available. Notably, the relevance of MDSCs in HIV-infected patients only began to be studied in 2012 (Vollbrecht et al., 2012; Qin et al., 2013). It has been reported that MDSCs positively correlate with viral load and negatively correlate with CD4+ T cell count (Dorhoi et al., 2020). Further supporting the involvement of MDSCs in HIV-infected persons, in 2014 a report described that gp120 HIV envelope protein was able to induce MDSC expansion from cultured peripheral blood mononuclear cells (PBMCs) (Garg and Spector, 2014). Simian immunodeficiency virus (SIV) is a retrovirus similar to HIV that belongs to the family of lentivirus. In 2015 a study performed on rhesus macaques described that the administration of a mix of several adjuvants alone (TLR agonists + IL-15 + other molecules) correlated with lower viral load after a SIV challenge as compared with immunization with several vaccine candidates (**Supplementary Table I**). Strikingly, all the vaccines tested caused increases in cells resembling MDSCs after immunization, whereas the treatment with the mix of adjuvants alone did not cause increases in those cells and correlated with better protection (Sui et al., 2014). According to these results, the authors suggested that preventing MDSC increases could be critical to improve vaccines against HIV (Sui et al., 2014).

Zhou, J. et al. (2013) developed a sPD1-p24fc/EP DNA DC-targeting vaccine, which induced a strong and durable HIV-1 GAG-specific CD8+ T lymphocyte response (Zhou et al., 2013). Interestingly, the same group found that after EcoHIV challenge in a murine model, the virus-infected and expanded MDSCs subverted the memory CD8+ T cell response at a very early stage of infection, allowing virus persistence, even in the context of a primed immune system (Liu et al., 2020). Moreover, depletion of MDSCs by anti-GR1 antibody administration (day 7, 9 and 11 post-infection) allowed a better clearance of infected cells, supporting that MDSCs diminished the efficacy of the vaccine (Liu et al., 2020).

Using a different vaccine candidate based on DNA-SIV+ALVAC-SIV+gp120 alum, it was observed that immunization of rhesus macaques increased the levels of M-MDSC-like cells (HLD-DR- CD14+), which was associated with an increased risk of SIV acquisition, and suggesting that MDSCs diminished vaccine effectiveness (Vaccari et al., 2019). In addition, the results supported the notion that an increased arginase activity linked to MDSCs may interfere with the proper induction of T and B cell responses. The authors highlighted the relevance of the understanding of the role of MDSCs to improve the efficacy of vaccine candidates, not only for HIV but also for other pathogens (Vaccari et al., 2019).

Interestingly, a mucosal vaccine against HIV/SIV in rhesus macaques also induced significant increases of both CD14+ M-MDSCs and CD15+ PMN-MDSCs in the PBMCs (Sui et al., 2019). The kinetic increase of the subsets was different, CD14+ MDSCs early increased from the first dose but then decreased progressively, whereas CD15+ PMN-MDSCs only increased significantly after the last dose of the vaccine regimen consisting of four doses. The authors suggested that both populations may be playing very different roles: CD15+ MDSCs were more likely to be early infected and suppressed the virus-specific T cell response; in contrast and strikingly, CD14+ M-MDSCs could be playing a protective role being part of the trained immunity (Sui et al., 2019). Based on these results, the authors suggest that induced trained immunity may be a new opportunity for innovative HIV vaccine design (Sui et al., 2019).

Although the study of the role of MDSCs in HIV infection started quite late, increasing evidence supports the notion that this population not only plays a major role in the pathogenesis of HIV infection, but also in the efficacy of a given vaccine against HIV (Dorhoi et al., 2020; Yaseen et al., 2021).

Using a late-stage vaccine study in rhesus macaques, it was reported that an influenza A vaccine consisting of modified RNAm induced M-MDSCs and PMN-MDSCs that shared several markers used to mark MDSCs in humans. Both subpopulations were able to suppress T cell proliferation *in vitro* and upregulated genes linked to MDSCs such as arginase-1, IDO1, PDL1, and IL-10 (Lin et al., 2018). Interestingly there are at least four vaccine studies performed in rhesus macaques showing that immunization induces MDSCs (2019; Sui et al., 2014; Lin et al., 2018; Vaccari et al., 2019). These results are important since late-stage studies in rhesus macaques are critical to test vaccines before clinical trials.

## MDSCs in vaccination against parasites

The involvement of MDSCs during *Leishmania* sp. infection has been demonstrated in several studies (Van Ginderachter et al., 2010; Pereira et al., 2011; Schmid et al., 2014). In 2015, immunization with a soluble leishmania antigen (SLA) from *L. donovani* increased spleen and liver MDSCs in a murine model. Interestingly, CD11b+ Gr-1+ cells from immunized mice were less suppressive than MDSCs induced by *L, donovani* infection (**Supplementary Table I**). Supporting these results, Cox-2, arginase-I, iNOS, and PGE2, were found to be less expressed in MDSCs from SLA-immunized mice (Bandyopadhyay et al., 2015).

In the same year, a malaria peptide antigen was assessed with different adjuvants in a murine model of vaccine development against *Plasmodium berghei* (Wilson et al., 2015). Strikingly, protective CD8 responses were obtained immunizing intradermically with the peptide linked to polystyrene nanoparticles (PSNP) or using Montanide as an adjuvant. Both approaches did not cause MDSC increases in the draining lymph node (DNL). In contrast, the use of Poli I:C as an adjuvant increased MDSCs in the DNL, a fact that correlated with the lack of CD8 protective response. This result is compatible with a scenario in which MDSCs may play a role in decreasing the efficacy of the vaccine adjuvanted with Poli I:C (Wilson et al., 2015). The authors also showed that administration of the adjuvants alone caused different ratios of GR-1+MDSCs/CD11c+ DCs. In this approach, only the Poly I:C adjuvant caused an increase in the ratio in the DNL supporting that this adjuvant generates a more suppressive environment as compared with Montanide or the nanoparticle-based adjuvant (Wilson et al., 2015). In a different work, the authors showed that a different vaccine adjuvanted with polystyrene nanoparticles was able to achieve partial protection against infection with blood-stage malaria (Wilson et al., 2019). These results suggest that further research using anti-inflammatory vaccine carriers and adjuvants may be valuable to improve malaria vaccines (Wilson et al., 2019).

The protozoan parasite *Trypanosoma cruzi (T. cruzi)* is the etiological agent of Chagas Disease (Pérez-Molina and Molina, 2018). Currently, there is substantial evidence supporting that acute parasite infection causes alterations in several immunoregulatory populations of the host, including MDSCs (Goñi et al., 2002; Arocena et al., 2014; Cardillo et al., 2015; Flávia Nardy et al., 2015; Poncini et al., 2015; Nardy et al., 2016; Prochetto et al., 2017). It has been reported that MDSCs increase in a very notable manner in the spleen, liver and heart of acutely infected mice (Goñi et al., 2002; Cuervo et al., 2011; Arocena et al., 2014). Only five years ago we reported that a Trans-sialidase-based vaccine was able to confer protective capacity against *T. cruzi* influencing not only the effector response but also the regulatory arm of the immune system (Prochetto et al., 2017). TSf-immunized mice had better survival against infection correlating with a significant decrease of spleen MDSCs as compared to non-immunized and infected mice (Prochetto et al., 2017). In another work, we observed that the remaining MDSCs in immunized and infected mice still have the capacity to strongly shape the immune response, since depletion of MDSCs with 5 fluorouracil (5FU) at day 15 post-infection in immunized mice caused a notable increase of the CD8 response and also affected dendritic cells and CD4+Foxp3+ regulatory T cells (Gamba et al., 2021). Interestingly, previous work had shown that depletion of MDSCs at day 10 or 15 post-infection, despite potentiating the immune response, also significantly increased the mortality to almost 100% in non-immunized mice (Arocena et al., 2014). Thus, the evidence suggests that MDSCs may be necessary to resolve inflammation in non-immunized mice, but vaccines may be able to allow decreases in MDSCs at the same time that increases the effector response and improve the outcome of the infection, including better survival, lower parasite and lower fibrosis in the heart of chronically infected mice (Prochetto et al., 2017; Gamba et al., 2021). The fact that MDSCs increase in a very notable manner during experimental acute *T. cruzi* infection, together with the fact that decreasing this population may notably reshape the immune response and affect the outcome of the infection, support the use of MDSCs as an important target to improve vaccine efficacy during rational vaccine design against *T. cruzi*.

## MDSCs in vaccination against fungi

Most of the studies addressing MDSCs in vaccination support the notion that those cells may play a role in decreasing the efficacy of the vaccine candidates. However, a protective role for MDSCs in vaccine studies has also been described. In this sense, it has been reported that intraperitoneal (i.p.) vaccination with low virulence/attenuated *Candida* species protects against i.p. or intravenous (i.v.) lethal *Candida albicans*/*Staphylococcus aureus* (Ca/Sa) challenge that causes sepsis in a murine model, (Lilly et al., 2019)(**Supplementary Table I**). The authors described that protection was long-lived (up to 60 days post-vaccination) and mediated by Ly6G+ Gr-1+ putative G-MDSCs, with no role for macrophages (Lilly et al., 2018; 2019; 2021). The adaptive immunity was not involved, since protection was maintained in RAG1^-/-^ mice lacking T and B cells. In addition, macrophages were neither involved in protection, since clodronate-mediated depletion of phagocytic macrophages failed to abrogate protection (Lilly et al., 2021).

Taking into consideration that macrophages have been involved in trained innate immunity (TII) (Ziogas and Netea, 2022), the authors proposed that Ly6G+ G-MDSCs may be considered a novel form of TII termed trained tolerogenic immunity (Lilly et al., 2021). It is worth mentioning that in a very different model of a mucosal vaccine against HIV infection of rhesus macaques, it has been proposed that CD14+ M-MDSCs could be playing a protective role being part of the trained immunity (Sui et al., 2019). Thus, both subtypes of MDSCs have been proposed to play protective roles after vaccination, as a part of innate trained immunity, in special contexts.

## MDSCs in adjuvant development

Adjuvants play a critical role in inducing and directing the immune response to improve vaccine efficacy (Awate et al., 2013; Apostólico et al., 2016).

Taking into account the studies collected for this review, it could be noted that MDSC induction is not an isolated event that occurs for one particular adjuvant. On the contrary, very different adjuvants were shown to cause MDSC increases. For instance, the studies performed in rhesus macaques, which is a critical late-stage vaccine testing model, showed that modified RNAm (Lin et al., 2018), DNA (Vaccari et al., 2019), and peptide-prime/MVA-SIV boost vaccines adjuvanted with IL-15, TLR agonists and other compounds (Sui et al., 2014), induced MDSCs. Modified RNAm and DNA were administered *via* intramuscular, whereas the other two studies using MVA-SIV vaccines were administered *via* intrarectal, supporting the concept that different routes of immunization and several adjuvants can induce MDSCs in rhesus macaques. Interestingly a study by Rosenbaum et al., 2021 analyzed whether the MVA vector inoculated by different routes influenced MDSCs in cynomolgus macaques (Rosenbaum et al., 2021). The authors described that all the immunization routes (intradermal, intramuscular, and subcutaneous) were associated with significant increases of CD14+ MDSCs and Lin^-^ MDSCs in the blood compartment. In addition, the recruitment of Lin^-^ MDSCs was greater after intramuscular immunization as compared with the other routes (Rosenbaum et al., 2021).

On the other hand, gastrointubation of attenuated *Salmonella* (Heithoff et al., 2008), or intradermal BCG immunization also induced MDSCs increases (Martino et al., 2010). Interestingly in 1982, it was reported that complete Freund adjuvant (CFA) emulsion that includes mycobacterium antigens affected the induction of suppressor T-cells in tumor-bearing hosts (Koyama et al., 1982). Further supporting this previous result, in 2010 it was also reported that CFA induced a population of myeloid cells with a suppressive capacity that was dependent on NO production (Wang et al., 2010). In addition, it has been recently described that subcutaneous immunization with CFA causes significant increases of MDSCs in the spleen of vaccinated mice (Ribechini et al., 2019). Intraperitoneal inoculation of CFA plus SLA antigen causes MDSC increases in a model used to assess a vaccine candidate against *Leishmania* spp. (Bandyopadhyay et al., 2015). Thus, substantial data is supporting the notion that attenuated pathogens or emulsified formulations of them can cause MDSC increases *via* another set of immunization routes such as gastrointubation, intradermal, intraperitoneal and subcutaneous immunization.

ISCOMs (immunostimulating complexes) are adjuvants composed of saponin, cholesterol, phospholipids and incorporated antigens (Morein et al., 1984). ISCOMATRIX and ISPA are similar adjuvants with cage-like particle structures but without an incorporated antigen (Bertona et al., 2017). We have previously shown that subcutaneous immunization with cage-like particles induces increases in CD11b+ GR-1+ cells resembling MDSCs (Prochetto et al., 2017). Moreover, in our model of *T. cruzi* infection, the pretreatment with 5FU in a dose reported by several groups to selectively deplete MDSCs (Vincent et al., 2010; Arocena et al., 2014; Namdar et al., 2015; Khosravianfar et al., 2018) allowed to significantly improve the protective capacity of a TSf-ISPA vaccine candidate allowing 100% of mice survival after a challenge with a lethal dose (Gamba et al., 2021).

Thus, there is wide evidence that different adjuvants and administration routes influence MDSCs in immunization protocols, suggesting that targeting those cells may be a valuable approach to improve vaccine efficacy against pathogens, as has been suggested (Gamba et al., 2021).

## Conditions that affect MDSCs also affect vaccination

Taking in mind that a substantial number of studies supports the notion that MDSCs may play a role in immunization against different pathogens, *via* different immunization routes and using different adjuvants, the question that arises is whether conditions that affect MDSCs would also influence vaccination. In the same line and further reinforcing the role of MDSCs in vaccination, it has been described that several conditions such as aging, obesity and newborns, which are known to affect MDSC levels, also influence vaccination.

The first study that addressed this topic was performed by Heithoff et al. (2008). In that work, in addition to showing that a *Salmonella* bivalent vaccine generated cross-protective capacity against *Salmonella*, it was also described that vaccinated aged mice, which had higher levels of MDSCs, exhibited a 50-fold increase of colony-forming unit (CFU) in the spleen relative to that of young mice. Similar results were obtained using both C57BL/6 and BALB/c mice, supporting that the observation is independent of the mouse strain (Heithoff et al., 2008). According to these results, the authors postulate that conditions capable of reducing MDSC numbers or activities in the elderly may be valuable to improve vaccine efficacy (Heithoff et al., 2008).

In a different approach but in the same line, Harman, et al. (2015) described that MDSCs expanded in aged mice after immunization with CpG-ODN emulsified with Freund´s adjuvant lasted longer in the spleen of aged mice than in their younger counterparts. CD11b+ Gr1+ cells highly express other surface proteins, such as CD124 and CD31, and were capable of suppressing T cell proliferative response by arginase induction (Harman et al., 2015).

Obesity is another condition that is associated with chronic inflammation and increases in MDSC basal levels (Ostrand-Rosenberg, 2018; Pawelec et al., 2019). In addition, obesity is a factor that correlates with a decreased vaccine-induced immune response (Painter et al., 2015). Using a murine model of obesity, vaccine efficacy against hepatitis B (HB) was tested. Interestingly, obese mice, which had increased proportions of MDSC in the spleen and liver, showed decreased humoral and HBsAg-specific cellular responses to the HB vaccine (Chen et al., 2015). Based on their results, the authors concluded that in their model obesity is related to impaired vaccine-induced humoral and cellular immunity (Chen et al., 2015).

It has been described that MDSCs present in newborns may be important to control inflammation (Gervassi et al., 2014; He et al., 2018; Köstlin-Gille and Gille, 2020). However, the presence of MDSCs may also be related to reduced response to vaccines in infants, as has been suggested (Gervassi et al., 2014). Recently, Kidzeru et al. (2021) analyzed MDSC frequencies in infants and immune responses to the vaccines Bacillus Calmette-Guérin (BCG), HB, and combination diphtheria, tetanus, and pertussis (dTaP). Higher MDSC frequency at vaccination was associated with a lack of subsequent IFN-ɣ release in response to vaccine Ags, except for BCG. No association was found between MDSCs and vaccine Ag-induced CD4+ T cell proliferative responses or humoral responses (Kidzeru et al., 2021). As suggested by the authors, the response to the vaccine may be affected by several factors such as vaccine composition, administration route, and capacity for inducing MDSCs as well as preexisting MDSC frequencies (Kidzeru et al., 2021).

The fact that MDSC levels are increased in very different conditions such as aging, obesity and newborns, in which a reduced vaccine response has been described, constitutes an additional line of evidence supporting that MDSCs may play an important role regarding vaccine efficacy. Moreover, it is very well-known that MDSCs are increased in several types of cancer and vaccine efficacy against cancer cells can be decreased by MDSCs (Poschke et al., 2012; Mengos et al., 2019; Shi et al., 2021).

## Concluding remarks

Vaccines are one of the most successful measures of public health to control diseases. Despite a substantial number of vaccines have been developed and included in vaccination schedules, there is still a long list of pathogens for which a licensed vaccine is still not available. Decades of research have not been sufficient to develop successful vaccines against complex pathogens such as HIV, HCV, *Plasmodium falciparum*, *Trypanosoma cruzi*, *Leishmania spp*, etc. Thus, the need for alternative strategies to improve vaccination has been mentioned in many of these cases (Koff et al., 2013).

One of the reasons that may account for the lacking vaccines could be related to the ability of the pathogens to evade and subvert the host immune system. In this sense, many pathogens can cause immunosuppression (Rueckert and Guzmán, 2012), a condition that may decrease not only the host response but also the response expected to be elicited by any vaccine candidate. Moreover, since vaccination aims to resemble certain aspects of the events that occur during natural infection, the immunization process by itself may expand populations of the regulatory arm of the immune system, such as MDSCs, decreasing vaccine efficacy. The reports collected in this review support the concept that induction of MDSCs during vaccination is not an isolated event that depends on a particular pathogen, adjuvant, or immunization route. On the contrary, consistent data support that induction of MDSCs is a very likely event that may take place during vaccination, although it is not generally assessed. If the vaccine by itself causes an expansion of MDSCs or other immunoregulatory populations, then the possibility that this vaccine cannot achieve its full potential exists, and targeting MDSCs may represent a valuable and promising tool to be explored as a strategy to improve the vaccine efficacy. For many pathogens, considering the effector arm of the immune response has been sufficient to develop successful vaccines. However, regarding the pathogens that precisely are endowed with the ability to subvert the host immune system, monitoring and/or targeting the MDSC population during rational vaccine design may constitute a valuable alternative to complement and further improve the vaccines that are still lacking.

## Author contributions

EP and EB wrote part of the manuscript and designed the Figures; CJ-C, VS-M, and GC planned the review, wrote some parts of the manuscript and revised the final version of the Figures and manuscript. All authors contributed to the article and approved the submitted version.

## Funding

This work was supported by ANPCyT (Argentine National Agency for the Promotion of Science and Technology) (PICT 2018-01164 and PICT 2019-01948), CONICET (National Scientific and Technical Research Council) and the Universidad Nacional del Litoral, Argentina.

## Conflict of interest

The authors declare that the research was conducted in the absence of any commercial or financial relationships that could be construed as a potential conflict of interest.

## Publisher’s note

All claims expressed in this article are solely those of the authors and do not necessarily represent those of their affiliated organizations, or those of the publisher, the editors and the reviewers. Any product that may be evaluated in this article, or claim that may be made by its manufacturer, is not guaranteed or endorsed by the publisher.

## References

[B1] Ahmadi M. Mohammadi M. Ali-Hassanzadeh M. Zare M. Gharesi-Fard B. (2019). MDSCs in pregnancy: Critical players for a balanced immune system at the feto-maternal interface. Cell Immunol. 346, 103990. doi: 10.1016/j.cellimm.2019.103990 31703912

[B2] al-Ramadi B. K. Chen Y. W. Meissler J. J. Eisenstein T. K. (1991). Immunosuppression induced by attenuated salmonella. reversal by IL-4. J. Immunol. 147, 1954–1961. 1909735

[B3] al-Ramadi B. K. Meissler J. J. Huang D. Eisenstein T. K. (1992). Immunosuppression induced by nitric oxide and its inhibition by interleukin-4. Eur. J. Immunol. 22, 2249–2254. doi: 10.1002/eji.1830220911 1516618

[B4] Apostólico J. de S. Lunardelli V. A. S. Coirada F. C. Boscardin S. B. Rosa D. S. (2016). Adjuvants: Classification, modus operandi, and licensing. J. Immunol. Res. 2016, e1459394. doi: 10.1155/2016/1459394 PMC4870346 27274998

[B5] Arocena A. R. Onofrio L. I. Pellegrini A. V. Carrera Silva A. E. Paroli A. Cano R. C.. (2014). Myeloid-derived suppressor cells are key players in the resolution of inflammation during a model of acute infection. Eur. J. Immunol. 44, 184–194. doi: 10.1002/eji.201343606 24166778

[B6] Awate S. Babiuk L. A. Mutwiri G. (2013). Mechanisms of action of adjuvants. Front. Immunol. 4. doi: 10.3389/fimmu.2013.00114 PMC3655441 23720661

[B7] Bandyopadhyay S. Bhattacharjee A. Banerjee S. Halder K. Das S. Paul Chowdhury B.. (2015). Glycyrrhizic acid-mediated subdual of myeloid-derived suppressor cells induces antileishmanial immune responses in a susceptible host. Infect. Immun. 83, 4476–4486. doi: 10.1128/IAI.00729-15 26351281 PMC4645397

[B8] Barreda D. R. Hanington P. C. Belosevic M. (2004). Regulation of myeloid development and function by colony stimulating factors. Dev. Comp. Immunol. 28, 509–554. doi: 10.1016/j.dci.2003.09.010 15062647

[B9] Bennett J. A. Rao V. S. Mitchell M. S. (1978). Systemic bacillus calmette-guérin (BCG) activates natural suppressor cells. Proc. Natl. Acad. Sci. U.S.A. 75, 5142–5144. doi: 10.1073/pnas.75.10.5142 283421 PMC336280

[B10] Bergenfelz C. Leandersson K. (2020). The generation and identity of human myeloid-derived suppressor cells. Front. Oncol. 10. doi: 10.3389/fonc.2020.00109 PMC7025543 32117758

[B11] Bertona D. Pujato N. Bontempi I. Gonzalez V. Cabrera G. Gugliotta L.. (2017). Development and assessment of a new cage-like particle adjuvant. J. Pharm. Pharmacol 10, 1293–1303. doi: 10.1111/jphp.12768 28664569

[B12] Blidner A. G. Méndez-Huergo S. P. Cagnoni A. J. Rabinovich G. A. (2015). Re-wiring regulatory cell networks in immunity by galectin–glycan interactions. FEBS Lett. 589, 3407–3418. doi: 10.1016/j.febslet.2015.08.037 26352298

[B13] Bronte V. Brandau S. Chen S.-H. Colombo M. P. Frey A. B. Greten T. F.. (2016). Recommendations for myeloid-derived suppressor cell nomenclature and characterization standards. Nat. Commun. 7, 12150. doi: 10.1038/ncomms12150 27381735 PMC4935811

[B14] Cabrera G. Marcipar I. (2019). Vaccines and the regulatory arm of the immune system. an overview from the trypanosoma cruzi infection model. Vaccine 37, 3628–3637. doi: 10.1016/j.vaccine.2019.05.015 31155420

[B15] Cardillo F. de Pinho R. T. Antas P. R. Z. Mengel J. (2015). Immunity and immune modulation in trypanosoma cruzi infection. Pathog. Dis. 73 (9), ftv082. doi: 10.1093/femspd/ftv082 26438729 PMC4626602

[B16] Cardona P. Cardona P.-J. (2019). Regulatory T cells in mycobacterium tuberculosis infection. Front. Immunol. 10, 2139. doi: 10.3389/fimmu.2019.02139 31572365 PMC6749097

[B17] Centuori S. M. Trad M. LaCasse C. J. Alizadeh D. Larmonier C. B. Hanke N. T.. (2012). Myeloid-derived suppressor cells from tumor-bearing mice impair TGF-β-induced differentiation of CD4+CD25+FoxP3+ tregs from CD4+CD25–FoxP3– T cells. J. Leukoc. Biol. 92, 987. doi: 10.1189/jlb.0911465 22891289 PMC3476240

[B18] Chen S. Akbar S. M. F. Miyake T. Abe M. Al-Mahtab M. Furukawa S.. (2015). Diminished immune response to vaccinations in obesity: role of myeloid-derived suppressor and other myeloid cells. Obes. Res. Clin. Pract. 9, 35–44. doi: 10.1016/j.orcp.2013.12.006 25660173

[B19] Condamine T. Mastio J. Gabrilovich D. I. (2015). Transcriptional regulation of myeloid-derived suppressor cells. J. Leukoc. Biol. 98, 913–922. doi: 10.1189/jlb.4RI0515-204R 26337512 PMC4661041

[B20] Cuervo H. Guerrero N. A. Carbajosa S. Beschin A. De Baetselier P. Gironès N.. (2011). Myeloid-derived suppressor cells infiltrate the heart in acute trypanosoma cruzi infection. J. Immunol. 187, 2656–2665. doi: 10.4049/jimmunol.1002928 21804013

[B21] Daker S. E. Sacchi A. Tempestilli M. Carducci C. Goletti D. Vanini V.. (2015). Granulocytic myeloid derived suppressor cells expansion during active pulmonary tuberculosis is associated with high nitric oxide plasma level. PloS One 10, e0123772. doi: 10.1371/journal.pone.0123772 25879532 PMC4400140

[B22] Davies L. C. Rice C. M. McVicar D. W. Weiss J. M. (2019). Diversity and environmental adaptation of phagocytic cell metabolism. J. Leukoc. Biol. 105, 37–48. doi: 10.1002/JLB.4RI0518-195R 30247792 PMC6334519

[B23] Deng J. Li J. Sarde A. Lines J. L. Lee Y.-C. Qian D. C.. (2019). Hypoxia-induced VISTA promotes the suppressive function of myeloid-derived suppressor cells in the tumor microenvironment. Cancer Immunol. Res. 7, 1079–1090. doi: 10.1158/2326-6066.CIR-18-0507 31088847 PMC6606337

[B24] De Wilde V. Van Rompaey N. Hill M. Lebrun J. F. Lemaître P. Lhommé F.. (2009). Endotoxin-induced myeloid-derived suppressor cells inhibit alloimmune responses *via* heme oxygenase-1. Am. J. Transplant. 9, 2034–2047. doi: 10.1111/j.1600-6143.2009.02757.x 19681826

[B25] Dietlin T. A. Hofman F. M. Lund B. T. Gilmore W. Stohlman S. A. van der Veen R. C. (2007). Mycobacteria-induced gr-1+ subsets from distinct myeloid lineages have opposite effects on T cell expansion. J. Leukoc. Biol. 81, 1205–1212. doi: 10.1189/jlb.1006640 17307863

[B26] Dietrich O. Heinz A. Goldmann O. Geffers R. Beineke A. Hiller K.. (2021). Dysregulated immunometabolism is associated with the generation of myeloid-derived suppressor cells in staphylococcus aureus chronic infection. J. Innate Immun. 14, 257–274d. doi: 10.1159/000519306 34763332 PMC9149459

[B27] Domínguez-Andrés J. van Crevel R. Divangahi M. Netea M. G. (2020). Designing the next generation of vaccines: Relevance for future pandemics. mBio 11, e02616–e02620. doi: 10.1128/mBio.02616-20 33443120 PMC8534290

[B28] Dorhoi A. Glaría E. Garcia-Tellez T. Nieuwenhuizen N. E. Zelinskyy G. Favier B.. (2019). MDSCs in infectious diseases: regulation, roles, and readjustment. Cancer Immunol. Immunother. 68, 673–685. doi: 10.1007/s00262-018-2277-y 30569204 PMC11028159

[B29] Dorhoi A. Kotzé L. A. Berzofsky J. A. Sui Y. Gabrilovich D. I. Garg A.. (2020). Therapies for tuberculosis and AIDS: myeloid-derived suppressor cells in focus. J. Clin. Invest. 130, 2789–2799. doi: 10.1172/JCI136288 32420917 PMC7260010

[B30] du Plessis N. Loebenberg L. Kriel M. von Groote-Bidlingmaier F. Ribechini E. Loxton A. G.. (2013). Increased frequency of myeloid-derived suppressor cells during active tuberculosis and after recent mycobacterium tuberculosis infection suppresses T-cell function. Am. J. Respir. Crit. Care Med. 188, 724–732. doi: 10.1164/rccm.201302-0249OC 23885784

[B31] Feikin D. R. Higdon M. M. Abu-Raddad L. J. Andrews N. Araos R. Goldberg Y.. (2022). Duration of effectiveness of vaccines against SARS-CoV-2 infection and COVID-19 disease: results of a systematic review and meta-regression. Lancet 399, 924–944. doi: 10.1016/S0140-6736(22)00152-0 35202601 PMC8863502

[B32] Flávia Nardy A. Freire-de-Lima C. G. Morrot A. (2015). Immune evasion strategies of trypanosoma cruzi. J. Immunol. Res. 2015, 178947. doi: 10.1155/2015/178947 26240832 PMC4512591

[B33] Fultang N. Li X. Li T. Chen Y. H. (2020). Myeloid-derived suppressor cell differentiation in cancer: Transcriptional regulators and enhanceosome-mediated mechanisms. Front. Immunol. 11, 619253. doi: 10.3389/fimmu.2020.619253 33519825 PMC7840597

[B34] Gabrilovich D. I. (2017). Myeloid-derived suppressor cells. Cancer Immunol. Res. 5, 3–8. doi: 10.1158/2326-6066.CIR-16-0297 28052991 PMC5426480

[B35] Gabrilovich D. I. Bronte V. Chen S.-H. Colombo M. P. Ochoa A. Ostrand-Rosenberg S.. (2007). The terminology issue for myeloid-derived suppressor cells. Cancer Res. 67, 425; author reply 426. doi: 10.1158/0008-5472.CAN-06-3037 17210725 PMC1941787

[B36] Gabrilovich D. I. Nagaraj S. (2009). Myeloid-derived suppressor cells as regulators of the immune system. Nat. Rev. Immunol. 9, 162–174. doi: 10.1038/nri2506 19197294 PMC2828349

[B37] Gabrilovich D. Nefedova Y. (2015). ROR1C regulates differentiation of myeloid-derived suppressor cells. Cancer Cell 28, 147–149. doi: 10.1016/j.ccell.2015.07.007 26267530 PMC4669045

[B38] Gamba J. C. Roldán C. Prochetto E. Lupi G. Bontempi I. Poncini C. V.. (2021). Targeting myeloid-derived suppressor cells to enhance a trans-Sialidase-Based vaccine against trypanosoma cruzi. Front. Cell. Infect. Microbiol. 11. doi: 10.3389/fcimb.2021.671104 PMC8290872 34295832

[B39] Garg A. Spector S. A. (2014). HIV Type 1 gp120–induced expansion of myeloid derived suppressor cells is dependent on interleukin 6 and suppresses immunity. J. Infect. Dis. 209, 441–451. doi: 10.1093/infdis/jit469 23999600 PMC3883171

[B40] Gershon R. K. Kondo K. (1970). Cell interactions in the induction of tolerance: the role of thymic lymphocytes. Immunology 18, 723–737. 4911896 PMC1455602

[B41] Gervassi A. Lejarcegui N. Dross S. Jacobson A. Itaya G. Kidzeru E.. (2014). Myeloid derived suppressor cells are present at high frequency in neonates and suppress *In vitro* T cell responses. PloS One 9, e107816. doi: 10.1371/journal.pone.0107816 25248150 PMC4172591

[B42] Goh C. Narayanan S. Hahn Y. S. (2013). Myeloid-derived suppressor cells: the dark knight or the joker in viral infections? Immunol. Rev. 255, 210–221. doi: 10.1111/imr.12084 23947357 PMC3748397

[B43] Goldblatt D. Alter G. Crotty S. Plotkin S. A. (2022). Correlates of protection against SARS-CoV-2 infection and COVID-19 disease. Immunol. Rev 10 (1), 6–26. doi: 10.1111/imr.13091 PMC9348242 35661178

[B44] Goñi O. Alcaide P. Fresno M. (2002). Immunosuppression during acute trypanosoma cruzi infection: involvement of Ly6G (Gr1(+))CD11b(+)immature myeloid suppressor cells. Int. Immunol. 14, 1125–1134. doi: 10.1093/intimm/dxf076 12356678

[B45] Hanson E. M. Clements V. K. Sinha P. Ilkovitch D. Ostrand-Rosenberg S. (2009). Myeloid-derived suppressor cells down-regulate l-selectin expression on CD4+ and CD8+ T cells. J. Immunol. 183, 937–944. doi: 10.4049/jimmunol.0804253 19553533 PMC2800824

[B46] Harman M. F. Ranocchia R. P. Gorlino C. V. Sánchez Vallecillo M. F. Castell S. D. Crespo M. I.. (2015). Expansion of myeloid-derived suppressor cells with arginase activity lasts longer in aged than in young mice after CpG-ODN plus IFA treatment. Oncotarget 6, 13448–13461. doi: 10.18632/oncotarget.3626 25922914 PMC4537026

[B47] Heithoff D. M. Enioutina E. Y. Bareyan D. Daynes R. A. Mahan M. J. (2008). Conditions that diminish myeloid-derived suppressor cell activities stimulate cross-protective immunity. Infect. Immun. 76, 5191–5199. doi: 10.1128/IAI.00759-08 18765736 PMC2573365

[B48] He Y.-M. Li X. Perego M. Nefedova Y. Kossenkov A. V. Jensen E. A.. (2018). Transitory presence of myeloid-derived suppressor cells in neonates is critical for control of inflammation. Nat. Med. 24, 224–231. doi: 10.1038/nm.4467 29334374 PMC5803434

[B49] Jayashankar L. Hafner R. (2016). Adjunct strategies for tuberculosis vaccines: Modulating key immune cell regulatory mechanisms to potentiate vaccination. Front. Immunol. 7. doi: 10.3389/fimmu.2016.00577 PMC5159487 28018344

[B50] Jiménez-Cortegana C. Sánchez-Jiménez F. Pérez-Pérez A. Álvarez N. Sousa A. Cantón-Bulnes L.. (2021). Low levels of granulocytic myeloid-derived suppressor cells may be a good marker of survival in the follow-up of patients with severe COVID-19. Front. Immunol. 12, 801410. doi: 10.3389/fimmu.2021.801410 35154077 PMC8835351

[B51] Ji J. Xu J. Zhao S. Liu F. Qi J. Song Y.. (2016). Myeloid-derived suppressor cells contribute to systemic lupus erythaematosus by regulating differentiation of Th17 cells and tregs. Clin. Sci. 130, 1453–1467. doi: 10.1042/CS20160311 27231253

[B52] Kelly A. M. McLoughlin R. M. (2020). Target the host, kill the bug; targeting host respiratory immunosuppressive responses as a novel strategy to improve bacterial clearance during lung infection. Front. Immunol. 11. doi: 10.3389/fimmu.2020.00767 PMC7203494 32425944

[B53] Khosravianfar N. Hadjati J. Namdar A. Boghozian R. Hafezi M. Ashourpour M.. (2018). Myeloid-derived suppressor cells elimination by 5-fluorouracil increased dendritic cell-based vaccine function and improved immunity in tumor mice. Iran J. Allergy Asthma Immunol. 17, 47–55. 29512369

[B54] Kidzeru E. Gasper M. A. Shao D. Edlefsen P. T. Lejarcegui N. Havyarimana E.. (2021). Myeloid-derived suppressor cells and their association with vaccine immunogenicity in south African infants. J. Leukoc. Biol. 110, 939–950. doi: 10.1002/JLB.5A0420-281R 33477200 PMC8292449

[B55] Knaul J. K. Jörg S. Oberbeck-Mueller D. Heinemann E. Scheuermann L. Brinkmann V.. (2014). Lung-residing myeloid-derived suppressors display dual functionality in murine pulmonary tuberculosis. Am. J. Respir. Crit. Care Med. 190, 1053–1066. doi: 10.1164/rccm.201405-0828OC 25275852

[B56] Koff W. C. Burton D. R. Johnson P. R. Walker B. D. King C. R. Nabel G. J.. (2013). Accelerating next-generation vaccine development for global disease prevention. Science 340, 1232910. doi: 10.1126/science.1232910 23723240 PMC4026248

[B57] Köstlin-Gille N. Gille C. (2020). Myeloid-derived suppressor cells in pregnancy and the neonatal period. Front. Immunol. 11. doi: 10.3389/fimmu.2020.584712 PMC7581934 33162999

[B58] Kotzé L. A. Young C. Leukes V. N. John V. Fang Z. Walzl G.. (2020). Mycobacterium tuberculosis and myeloid-derived suppressor cells: Insights into caveolin rich lipid rafts. EBioMedicine 53, 102670. doi: 10.1016/j.ebiom.2020.102670 32113158 PMC7047144

[B59] Koushki K. Salemi M. Miri S. M. Arjeini Y. Keshavarz M. Ghaemi A. (2021). Role of myeloid-derived suppressor cells in viral respiratory infections; hints for discovering therapeutic targets for COVID-19. BioMed. Pharmacother. 144, 112346. doi: 10.1016/j.biopha.2021.112346 34678727 PMC8516725

[B60] Koyama S. Yoshioka T. Sakita T. (1982). Suppression of cell-mediated antitumor immunity by complete freund’s adjuvant. Cancer Res. 42, 3215–3219. 6980047

[B61] Kusmartsev S. Nagaraj S. Gabrilovich D. I. (2005). Tumor-associated CD8+ T cell tolerance induced by bone marrow-derived immature myeloid cells. J. Immunol. 175, 4583–4592. doi: 10.4049/jimmunol.175.7.4583 16177103 PMC1350970

[B62] Li H. Han Y. Guo Q. Zhang M. Cao X. (2009). Cancer-expanded myeloid-derived suppressor cells induce anergy of NK cells through membrane-bound TGF-beta 1. J. Immunol. 182, 240–249. doi: 10.4049/jimmunol.182.1.240 19109155

[B63] Lilly E. A. Bender B. E. Esher Righi S. Fidel P. L. Noverr M. C. (2021). Trained innate immunity induced by vaccination with low-virulence candida species mediates protection against several forms of fungal sepsis *via* Ly6G+ gr-1+ leukocytes. mBio 12, e0254821. doi: 10.1128/mBio.02548-21 34663098 PMC8524338

[B64] Lilly E. A. Ikeh M. Nash E. E. Fidel P. L. Noverr M. C. (2018). Immune protection against lethal fungal-bacterial intra-abdominal infections. mBio 9, e01472-17. doi: 10.1128/mBio.01472-17 29339423 PMC5770546

[B65] Lilly E. A. Yano J. Esher S. K. Hardie E. Fidel P. L. Noverr M. C. (2019). Spectrum of trained innate immunity induced by low-virulence candida species against lethal polymicrobial intra-abdominal infection. Infect. Immun. 87, e00348-19. doi: 10.1128/IAI.00348-19 31085710 PMC6652762

[B66] Lin A. Liang F. Thompson E. A. Vono M. Ols S. Lindgren G.. (2018). Rhesus macaque myeloid-derived suppressor cells demonstrate T cell inhibitory functions and are transiently increased after vaccination. J. Immunol. 200, 286–294. doi: 10.4049/jimmunol.1701005 29180488

[B67] Li K. Shi H. Zhang B. Ou X. Ma Q. Chen Y.. (2021). Myeloid-derived suppressor cells as immunosuppressive regulators and therapeutic targets in cancer. Signal Transduct Target Ther. 6, 362. doi: 10.1038/s41392-021-00670-9 34620838 PMC8497485

[B68] Liu L. Lin Q. Peng J. Fang J. Tan Z. Tang H.. (2020). Persistent lentivirus infection induces early myeloid suppressor cells expansion to subvert protective memory CD8 T cell response✰,✰✰. EBioMedicine 60, 103008. doi: 10.1016/j.ebiom.2020.103008 32979832 PMC7519271

[B69] Loftus T. J. Mohr A. M. Moldawer L. L. (2018). Dysregulated myelopoiesis and hematopoietic function following acute physiologic insult. Curr. Opin. Hematol. 25, 37–43. doi: 10.1097/MOH.0000000000000395 29035909 PMC5733709

[B70] Lu C. Redd P. S. Lee J. R. Savage N. Liu K. (2016). The expression profiles and regulation of PD-L1 in tumor-induced myeloid-derived suppressor cells. Oncoimmunology 5, e1247135. doi: 10.1080/2162402X.2016.1247135 28123883 PMC5214087

[B71] Magcwebeba T. Dorhoi A. du Plessis N. (2019) The emerging role of myeloid-derived suppressor cells in tuberculosis. frontiers in immunology. Available at: https://www.frontiersin.org/article/10.3389/fimmu.2019.00917 (Accessed March 5, 2022). 10.3389/fimmu.2019.00917 PMC6502992 31114578

[B72] Martino A. Badell E. Abadie V. Balloy V. Chignard M. Mistou M.-Y.. (2010). Mycobacterium bovis bacillus calmette-guérin vaccination mobilizes innate myeloid-derived suppressor cells restraining *in vivo* T cell priming *via* IL-1R-dependent nitric oxide production. J. Immunol. 184, 2038–2047. doi: 10.4049/jimmunol.0903348 20083674

[B73] Medina E. Hartl D. (2018). Myeloid-derived suppressor cells in infection: A general overview. J. Innate Immun. 10, 407–413. doi: 10.1159/000489830 29945134 PMC6784037

[B74] Mengos A. E. Gastineau D. A. Gustafson M. P. (2019). The CD14+HLA-DRlo/neg monocyte: An immunosuppressive phenotype that restrains responses to cancer immunotherapy. Front. Immunol. 10. doi: 10.3389/fimmu.2019.01147 PMC6540944 31191529

[B75] Millrud C. R. Bergenfelz C. Leandersson K. (2016). On the origin of myeloid-derived suppressor cells. Oncotarget 8, 3649–3665. doi: 10.18632/oncotarget.12278 PMC5356220 27690299

[B76] Milne G. Hames T. Scotton C. Gent N. Johnsen A. Anderson R. M.. (2021). Does infection with or vaccination against SARS-CoV-2 lead to lasting immunity? Lancet Respir. Med. 9, 1450–1466. doi: 10.1016/S2213-2600(21)00407-0 34688434 PMC8530467

[B77] Mitchell M. S. Murahata R. I. (1979). Modulation of immunity by bacillus calmette-guérin (BCG). Pharmacol. Ther. 4, 329–353. doi: 10.1016/0163-7258(79)90141-4 386385

[B78] Montagnier L. (2010). 25 years after HIV discovery: Prospects for cure and vaccine. Virology 397, 248–254. doi: 10.1016/j.virol.2009.10.045 20152474

[B79] Morein B. Sundquist B. Höglund S. Dalsgaard K. Osterhaus A. (1984). Iscom, a novel structure for antigenic presentation of membrane proteins from enveloped viruses. Nature 308, 457–460. doi: 10.1038/308457a0 6709052

[B80] Nagaraj S. Gabrilovich D. I. (2007). Myeloid-derived suppressor cells. Adv. Exp. Med. Biol. 601, 213–223. doi: 10.1007/978-0-387-72005-0_22 17713008

[B81] Nagaraj S. Nelson A. Youn J. Cheng P. Quiceno D. Gabrilovich D. I. (2012). Antigen-specific CD4(+) T cells regulate function of myeloid-derived suppressor cells in cancer *via* retrograde MHC class II signaling. Cancer Res. 72, 928–938. doi: 10.1158/0008-5472.CAN-11-2863 22237629 PMC4062074

[B82] Namdar A. Mirzaei H. R. Jadidi-Niaragh F. Ashourpour M. Ajami M. Hadjati J.. (2015). Multiple low doses of 5-fluorouracil diminishes immunosuppression by myeloid derived suppressor cells in murine melanoma model. Iran J. Immunol. 12, 176–187 26412636 10.22034/iji.2015.16747

[B83] Nam S. Lee A. Lim J. Lim J.-S. (2019). Analysis of the expression and regulation of PD-1 protein on the surface of myeloid-derived suppressor cells (MDSCs). Biomol. Ther. (Seoul) 27, 63–70. doi: 10.4062/biomolther.2018.201 30521746 PMC6319546

[B84] Nardy A. F. F. R. Freire-de-Lima C. G. Pérez A. R. Morrot A. (2016). Role of trypanosoma cruzi trans-sialidase on the escape from host immune surveillance. Front. Microbiol. 7. doi: 10.3389/fmicb.2016.00348 PMC4804232 27047464

[B85] O’Connor M. A. Rastad J. L. Green W. R. (2017). The role of myeloid-derived suppressor cells in viral infection. Viral Immunol. 30, 82–97. doi: 10.1089/vim.2016.0125 28051364 PMC5346953

[B86] Ostrand-Rosenberg S. (2018). Myeloid derived-suppressor cells: their role in cancer and obesity. Curr. Opin. Immunol. 51, 68–75. doi: 10.1016/j.coi.2018.03.007 29544121 PMC5943167

[B87] Ostrand-Rosenberg S. Fenselau C. (2018). Myeloid-derived suppressor cells: Immune-suppressive cells that impair antitumor immunity and are sculpted by their environment. J. Immunol. 200, 422–431. doi: 10.4049/jimmunol.1701019 29311384 PMC5765878

[B88] Ost M. Singh A. Peschel A. Mehling R. Rieber N. Hartl D. (2016). Myeloid-derived suppressor cells in bacterial infections. Front. Cell Infect. Microbiol. 6. doi: 10.3389/fcimb.2016.00037 PMC4814452 27066459

[B89] Painter S. D. Ovsyannikova I. G. Poland G. A. (2015). The weight of obesity on the human immune response to vaccination. Vaccine 33, 4422–4429. doi: 10.1016/j.vaccine.2015.06.101 26163925 PMC4547886

[B90] Pan P.-Y. Ma G. Weber K. J. Ozao-Choy J. Wang G. Yin B.. (2010). Immune stimulatory receptor CD40 is required for T-cell suppression and T regulatory cell activation mediated by myeloid-derived suppressor cells in cancer. Cancer Res. 70, 99–108. doi: 10.1158/0008-5472.CAN-09-1882 19996287 PMC2805053

[B91] Panopoulos A. D. Watowich S. S. (2008). GRANULOCYTE COLONY-STIMULATING FACTOR: MOLECULAR MECHANISMS OF ACTION DURING STEADY STATE AND ‘EMERGENCY’ HEMATOPOIESIS. Cytokine 42, 277–288. doi: 10.1016/j.cyto.2008.03.002 18400509 PMC2852428

[B92] Parker K. H. Sinha P. Horn L. A. Clements V. K. Yang H. Li J.. (2014). HMGB1 enhances immune suppression by facilitating the differentiation and suppressive activity of myeloid-derived suppressor cells. Cancer Res. 74, 5723–5733. doi: 10.1158/0008-5472.CAN-13-2347 25164013 PMC4199911

[B93] Pawelec G. Picard E. Bueno V. Verschoor C. P. Ostrand-Rosenberg S. (2021). MDSCs, ageing and inflammageing. Cell. Immunol. 362, 104297. doi: 10.1016/j.cellimm.2021.104297 33550187

[B94] Pawelec G. Verschoor C. P. Ostrand-Rosenberg S. (2019). Myeloid-derived suppressor cells: Not only in tumor immunity. Front. Immunol. 10. doi: 10.3389/fimmu.2019.01099 PMC6529572 31156644

[B95] Pereira W. F. Ribeiro-Gomes F. L. Guillermo L. V. C. Vellozo N. S. Montalvão F. Dosreis G. A.. (2011). Myeloid-derived suppressor cells help protective immunity to leishmania major infection despite suppressed T cell responses. J. Leukoc. Biol. 90, 1191–1197. doi: 10.1189/jlb.1110608 21934068

[B96] Pérez-Molina J. A. Molina I. (2018). Chagas disease. Lancet 391, 82–94. doi: 10.1016/S0140-6736(17)31612-4 28673423

[B97] Plitas G. Rudensky A. Y. (2016). Regulatory T cells: Differentiation and function. Cancer Immunol. Res. 4, 721–725. doi: 10.1158/2326-6066.CIR-16-0193 27590281 PMC5026325

[B98] Plotkin S. A. (2018). Vaccines we need but don’t have. Viral Immunol. 31, 114–116. doi: 10.1089/vim.2017.0126 29131713

[B99] Plotkin S. A. (2020). Updates on immunologic correlates of vaccine-induced protection. Vaccine 38, 2250–2257. doi: 10.1016/j.vaccine.2019.10.046 31767462

[B100] Pollard A. J. Bijker E. M. (2021). A guide to vaccinology: from basic principles to new developments. Nat. Rev. Immunol. 21, 83–100. doi: 10.1038/s41577-020-00479-7 33353987 PMC7754704

[B101] Poncini C. V. Ilarregui J. M. Batalla E. I. Engels S. Cerliani J. P. Cucher M. A.. (2015). Trypanosoma cruzi infection imparts a regulatory program in dendritic cells and T cells *via* galectin-1-Dependent mechanisms. J. Immunol. 195, 3311–3324. doi: 10.4049/jimmunol.1403019 26324777

[B102] Poschke I. Mao Y. Adamson L. Salazar-Onfray F. Masucci G. Kiessling R. (2012). Myeloid-derived suppressor cells impair the quality of dendritic cell vaccines. Cancer Immunol. Immunother. 61, 827–838. doi: 10.1007/s00262-011-1143-y 22080405 PMC11028420

[B103] Prochetto E. Roldán C. Bontempi I. A. Bertona D. Peverengo L. Vicco M. H.. (2017). Trans-sialidase-based vaccine candidate protects against trypanosoma cruzi infection, not only inducing an effector immune response but also affecting cells with regulatory/suppressor phenotype. Oncotarget 8 (35), 58003–58020. doi: 10.18632/oncotarget.18217 28938533 PMC5601629

[B104] Qin A. Cai W. Pan T. Wu K. Yang Q. Wang N.. (2013). Expansion of monocytic myeloid-derived suppressor cells dampens T cell function in HIV-1-seropositive individuals. J. Virol. 87, 1477–1490. doi: 10.1128/JVI.01759-12 23152536 PMC3554138

[B105] Qu P. Boelte K. C. Charles Lin P. (2012). Negative regulation of myeloid-derived suppressor cells in cancer. Immunol. Invest. 41, 562–580. doi: 10.3109/08820139.2012.685538 23017135 PMC7469827

[B106] Raber P. L. Thevenot P. Sierra R. Wyczechowska D. Halle D. Ramirez M. E.. (2014). Subpopulations of myeloid-derived suppressor cells impair T cell responses through independent nitric oxide-related pathways. Int. J. Cancer 134, 2853–2864. doi: 10.1002/ijc.28622 24259296 PMC3980009

[B107] Ribechini E. Eckert I. Beilhack A. Du Plessis N. Walzl G. Schleicher U.. (2019). Heat-killed mycobacterium tuberculosis prime-boost vaccination induces myeloid-derived suppressor cells with spleen dendritic cell-killing capability. JCI Insight 4 (13), e128664. doi: 10.1172/jci.insight.128664 PMC6629241 31162143

[B108] Rosenbaum P. Tchitchek N. Joly C. Rodriguez Pozo A. Stimmer L. Langlois S.. (2021). Vaccine inoculation route modulates early immunity and consequently antigen-specific immune response. Front. Immunol. 12. doi: 10.3389/fimmu.2021.645210 PMC8093451 33959127

[B109] Rueckert C. Guzmán C. A. (2012). Vaccines: From empirical development to rational design. PloS Pathog. 8, e1003001. doi: 10.1371/journal.ppat.1003001 23144616 PMC3493475

[B110] Ryzhov S. Novitskiy S. V. Goldstein A. E. Biktasova A. Blackburn M. R. Biaggioni I.. (2011). Adenosinergic regulation of the expansion and immunosuppressive activity of CD11b+Gr1+ cells. J. Immunol. 187, 6120–6129. doi: 10.4049/jimmunol.1101225 22039302 PMC3221925

[B111] Sakaguchi S. Sakaguchi N. Asano M. Itoh M. Toda M. (1995). Immunologic self-tolerance maintained by activated T cells expressing IL-2 receptor alpha-chains (CD25). breakdown of a single mechanism of self-tolerance causes various autoimmune diseases. J. Immunol. 155, 1151–1164. 7636184

[B112] Sakaguchi S. Wing K. Miyara M. (2007). Regulatory T cells - a brief history and perspective. Eur. J. Immunol. 37 (Suppl 1), 116–123. doi: 10.1002/eji.200737593 17972355

[B113] Schaible U. E. Linnemann L. Redinger N. Patin E. C. Dallenga T. (2017). Strategies to improve vaccine efficacy against tuberculosis by targeting innate immunity. Front. Immunol. 8. doi: 10.3389/fimmu.2017.01755 PMC5732265 29312298

[B114] Schlecker E. Stojanovic A. Eisen C. Quack C. Falk C. S. Umansky V.. (2012). Tumor-infiltrating monocytic myeloid-derived suppressor cells mediate CCR5-dependent recruitment of regulatory T cells favoring tumor growth. J. Immunol. 189, 5602–5611. doi: 10.4049/jimmunol.1201018 23152559

[B115] Schmid M. Zimara N. Wege A. K. Ritter U. (2014). Myeloid-derived suppressor cell functionality and interaction with leishmania major parasites differ in C57BL/6 and BALB/c mice. Eur. J. Immunol. 44, 3295–3306. doi: 10.1002/eji.201344335 25142017

[B116] Shevach E. M. (2006). From vanilla to 28 flavors: multiple varieties of T regulatory cells. Immunity 25, 195–201. doi: 10.1016/j.immuni.2006.08.003 16920638

[B117] Shi H. Li K. Ni Y. Liang X. Zhao X. (2021). Myeloid-derived suppressor cells: Implications in the resistance of malignant tumors to T cell-based immunotherapy. Front. Cell Dev. Biol. 9. doi: 10.3389/fcell.2021.707198 PMC8317971 34336860

[B118] Singh A. Lelis F. Braig S. Schäfer I. Hartl D. Rieber N. (2016) Differential regulation of myeloid-derived suppressor cells by candida species. frontiers in microbiology. Available at: https://www.frontiersin.org/article/10.3389/fmicb.2016.01624 (Accessed March 6, 2022). 10.3389/fmicb.2016.01624 PMC5061774 27790210

[B119] Solito S. Pinton L. De Sanctis F. Ugel S. Bronte V. Mandruzzato S.. (2019). Methods to measure MDSC immune suppressive activity *In vitro* and *In vivo* . Curr. Protoc. Immunol. 124, e61. doi: 10.1002/cpim.61 30303619

[B120] Song X. Krelin Y. Dvorkin T. Bjorkdahl O. Segal S. Dinarello C. A.. (2005). CD11b+/Gr-1+ immature myeloid cells mediate suppression of T cells in mice bearing tumors of IL-1beta-secreting cells. J. Immunol. 175, 8200–8208. doi: 10.4049/jimmunol.175.12.8200 16339559

[B121] Srivastava M. K. Sinha P. Clements V. K. Rodriguez P. Ostrand-Rosenberg S. (2010). Myeloid-derived suppressor cells inhibit T-cell activation by depleting cystine and cysteine. Cancer Res. 70, 68–77. doi: 10.1158/0008-5472.CAN-09-2587 20028852 PMC2805057

[B122] Sui Y. Hogg A. Wang Y. Frey B. Yu H. Xia Z.. (2014). Vaccine-induced myeloid cell population dampens protective immunity to SIV. J. Clin. Invest. 124, 2538–2549. doi: 10.1172/JCI73518 24837435 PMC4038576

[B123] Sui Y. Lewis G. K. Wang Y. Berckmueller K. Frey B. Dzutsev A.. (2019). Mucosal vaccine efficacy against intrarectal SHIV is independent of anti-env antibody response. J. Clin. Invest. 129, 1314–1328. doi: 10.1172/JCI122110 30776026 PMC6391089

[B124] Takizawa H. Boettcher S. Manz M. G. (2012). Demand-adapted regulation of early hematopoiesis in infection and inflammation. Blood 119, 2991–3002. doi: 10.1182/blood-2011-12-380113 22246037

[B125] Talmadge J. E. Gabrilovich D. I. (2013). History of myeloid derived suppressor cells (MDSCs) in the macro- and micro-environment of tumour-bearing hosts. Nat. Rev. Cancer 13, 739–752. doi: 10.1038/nrc3581 24060865 PMC4358792

[B126] Ugolini A. Tyurin V. A. Tyurina Y. Y. Tcyganov E. N. Donthireddy L. Kagan V. E.. (2020). Polymorphonuclear myeloid-derived suppressor cells limit antigen cross-presentation by dendritic cells in cancer. JCI Insight 5, 138581. doi: 10.1172/jci.insight.138581 32584791 PMC7455061

[B127] Vaccari M. Fourati S. Brown D. R. Silva de Castro I. Bissa M. Schifanella L.. (2019). Myeloid cell crosstalk regulates the efficacy of the DNA/ALVAC/gp120 HIV vaccine candidate. Front. Immunol. 10, 1072. doi: 10.3389/fimmu.2019.01072 31139193 PMC6527580

[B128] Van Ginderachter J. A. Beschin A. De Baetselier P. Raes G. (2010). Myeloid-derived suppressor cells in parasitic infections. Eur. J. Immunol. 40, 2976–2985. doi: 10.1002/eji.201040911 21061431

[B129] Veglia F. Perego M. Gabrilovich D. (2018). Myeloid-derived suppressor cells coming of age. Nat. Immunol. 19, 108–119. doi: 10.1038/s41590-017-0022-x 29348500 PMC5854158

[B130] Veglia F. Sanseviero E. Gabrilovich D. I. (2021). Myeloid-derived suppressor cells in the era of increasing myeloid cell diversity. Nat. Rev. Immunol. 21, 485–498. doi: 10.1038/s41577-020-00490-y 33526920 PMC7849958

[B131] Vincent J. Mignot G. Chalmin F. Ladoire S. Bruchard M. Chevriaux A.. (2010). 5-fluorouracil selectively kills tumor-associated myeloid-derived suppressor cells resulting in enhanced T cell-dependent antitumor immunity. Cancer Res. 70, 3052–3061. doi: 10.1158/0008-5472.CAN-09-3690 20388795

[B132] Vollbrecht T. Stirner R. Tufman A. Roider J. Huber R. M. Bogner J. R.. (2012). Chronic progressive HIV-1 infection is associated with elevated levels of myeloid-derived suppressor cells. AIDS 26, F31–F37. doi: 10.1097/QAD.0b013e328354b43f 22526518

[B133] Wang Z. Jiang J. Li Z. Zhang J. Wang H. Qin Z. (2010). A myeloid cell population induced by freund adjuvant suppresses T-cell-mediated antitumor immunity. J. Immunother. 33, 167–177. doi: 10.1097/CJI.0b013e3181bed2ba 20145547

[B134] Wilson K. L. Pouniotis D. Hanley J. Xiang S. D. Ma C. Coppel R. L.. (2019) A synthetic nanoparticle based vaccine approach targeting MSP4/5 is immunogenic and induces moderate protection against murine blood-stage malaria. frontiers in immunology. Available at: https://www.frontiersin.org/article/10.3389/fimmu.2019.00331 (Accessed May 17, 2022). 10.3389/fimmu.2019.00331 PMC6428706 30930890

[B135] Wilson K. L. Xiang S. D. Plebanski M. (2015). Montanide, poly I:C and nanoparticle based vaccines promote differential suppressor and effector cell expansion: a study of induction of CD8 T cells to a minimal plasmodium berghei epitope. Front. Microbiol. 6. doi: 10.3389/fmicb.2015.00029 PMC4319470 25705207

[B136] Yang R. Cai Z. Zhang Y. Yutzy W. H. Roby K. F. Roden R. B. S. (2006). CD80 in immune suppression by mouse ovarian carcinoma–associated gr-1+CD11b+ myeloid cells. Cancer Res. 66, 6807–6815. doi: 10.1158/0008-5472.CAN-05-3755 16818658

[B137] Yang Y. Li C. Liu T. Dai X. Bazhin A. V. (2020) Myeloid-derived suppressor cells in tumors: From mechanisms to antigen specificity and microenvironmental regulation. frontiers in immunology. Available at: https://www.frontiersin.org/article/10.3389/fimmu.2020.01371 (Accessed February 10, 2022). 10.3389/fimmu.2020.01371 PMC7387650 32793192

[B138] Yaseen M. M. Abuharfeil N. M. Darmani H. (2021). The impact of MDSCs on the efficacy of preventive and therapeutic HIV vaccines. Cell Immunol. 369, 104440. doi: 10.1016/j.cellimm.2021.104440 34560382

[B139] Zhou J. Cheung A. K. L. Tan Z. Wang H. Yu W. Du Y.. (2013). PD1-based DNA vaccine amplifies HIV-1 GAG-specific CD8^+^ T cells in mice. J. Clin. Invest. 123, 2629–2642. doi: 10.1172/JCI64704 23635778 PMC3668817

[B140] Ziogas A. Netea M. G. (2022). Trained immunity-related vaccines: innate immune memory and heterologous protection against infections. Trends Mol. Med. 28 (6), P497–512. doi: 10.1016/j.molmed.2022.03.009 35466062

