# Supplementary material for: Myeloid-derived suppressor cells and vaccination against pathogens
Source: Front Cell Infect Microbiol. 2022 Sep 29;12:1003781. doi: 10.3389/fcimb.2022.1003781 (PMC9557202; doi:10.3389/fcimb.2022.1003781)
Supplement: Supplementary file 1 [file DataSheet_1.pdf]

Table I. Immunization against pathogens and MDSC related observations

Type of Pathogen	Immunization	Animal model	Vaccine Administration Route	Observation	Reference
Bacteria .	BCG	Mice	Intravenous	Natural suppressor cells were activated and increased in the spleen after BCG administration	(Bennett et al., 1978)
	BCG	Mice	Intradermal	NO-producing MDSCs with ability to impair T cell priming were recruited to the site of inoculation	(Martino et al., 2010)
	M. Tuberculosis in CFA	Mice	Subcutaneous	Gr-1+ splenocytes from primed mice showed suppressive capacity in vitro	(Dietlin et al., 2007)
	M. Tuberculosis in CFA.	Mice	Subcutaneous	CD11b+ Gr-1+ cells with NO-dependent suppressive capacity increased in the spleen	(Ribechini et al., 2019)
	Mycobacterium tuberculosis in CFA plus LPS/IFN-γ	Mice	Subcutaneous	M-MDSCs with ability to kill dendritic cells (DCs) were found in the spleen	(Ribechini et al., 2019)
	Attenuated *Salmonella typhimurium*	Mice	Intraperitoneal	Immunization induced NO-producing macrophage precursors with suppressive capacity	(al-Ramadi et al., 1991) (al-Ramadi et al., 1992)
	Salmonella bivalent vaccine	Mice	Orally by gastrointubation	Lower levels of MDSCs in immunized and orally infected mice correlated with increased humoral and cellular immune response	(Heithoff et al., 2008)

Viruses	Mix of several adjuvants	Rhesus macaques	Intracolorectal	Lower levels of MDSC resembling cells in adjuvant treated animals correlated with lower viral load after a SIV challenge.	(Sui et al., 2014)
	PD1-based vaccine	Mice	Intramuscular	EcoHIV i.p. infection expanded MDSCs. Depletion of MDSCs allowed a better clearance of infected cells	(Liu et al., 2020)
	DNA-SIV+ALVAC-SIV + gp120 alum	Rhesus macaques	Intramuscular	Immunized macaques increased the levels of M-MDSC-like cells and had increased risk of SIV infection.	(Vaccari et al., 2019)
	HIV/SIV peptides, + MVA-SIV antigens, + HIVgp120–CD4 fusion protein.	Rhesus macaques	Intrarectal	After intrarectal SIV challenge, vaccinated animals had significant increases of CD14+ M-MDSCs and CD15+ PMN-MDSCs in the PBMCs.	(Sui et al., 2019)
	mRNA encoding the hemagglutinin of H10N8 influenza A virus.	Rhesus macaques	Intramuscular	Immunization induced M-MDSCs and PMN-MDSCs with ability to suppress T cell proliferation in vitro.	(Lin et al., 2018)
Fungi	Low-virulence *Candida* strains.	Mice	Intraperitoneal or intravenous	Immunized mice increased MDSCs in the peritoneal cavity after a challenge with Ca/Sa. Depletion of Ly6G+ cells increased mortality of vaccinated animals	(LillY et al., 2018). (Lilly et al., 2021)

Parasites	*SLA from L. donovani.*	Mice	Intraperitoneal	Immunization increased spleen and liver MDSCs that were less suppressive than MDSCs induced by *L. donovani* infection	(Bandyopadhyay et al., 2015)
	Peptide linked to PSNP, or peptide formulated with Montanide or Poli I:C as adjuvants.	Mice	Intradermical	Immunization using PSNP or Montanide did not cause MDSC increases and generated a CD8 response against P. berghei. In contrast, Poli I:C increased MDSCs, correlating with lack of CD8 protective response	(Wilson et al., 2015)
	TSf from *T. cruzi* formulated with ISPA.	Mice	Subcutaneous	TSf-ISPA immunized mice had better survival against *T. cruzi*, correlating with a significant decrease of spleen MDSCs as compared to non-immunized and infected mice	(Prochetto et al., 2017). (Gamba et al., 2021)

Table legend: *M. tuberculosis*: *Mycobacterium tuberculosis*. CFA: Complete Freund Adjuvant; *S. Typhimurium: Salmonella Typhimurium*; i.p.: intraperitoneal; MVA: Modified vaccinia Ankara; SLA: Soluble *Leishmania* antigen; *L. donovani*: *Leishmania Donovani*; PSNP: polystyrene nanoparticles; *P. berghei*: *Plasmodium berghei*; TSf: Trans-sialidase fragment; *T. cruzi*: *Trypanosoma cruzi*; ISPA: immunostimulating cage like particles; Ca/Sa: *Candida albicans/Staphylococcus aureus.*
